# Toll-Like Receptor Ligand-Based Vaccine Adjuvants Require Intact MyD88 Signaling in Antigen-Presenting Cells for Germinal Center Formation and Antibody Production

**DOI:** 10.3389/fimmu.2017.00225

**Published:** 2017-03-03

**Authors:** Munir M. Mosaheb, Michael L. Reiser, Lee M. Wetzler

**Affiliations:** ^1^Department of Microbiology, Boston University School of Medicine, Boston, MA, USA; ^2^Department of Medicine, Section of Infectious Diseases, Boston Medical Center, Boston, MA, USA

**Keywords:** vaccines, MyD88, toll-like receptors, germinal centers, adjuvants, *Neisseria meningitidis*, porin B

## Abstract

Vaccines are critical in the fight against infectious diseases, and immune-stimulating adjuvants are essential for enhancing vaccine efficacy. However, the precise mechanisms of action of most adjuvants are unknown. There is an urgent need for customized and adjuvant formulated vaccines against immune evading pathogens that remain a risk today. Understanding the specific role of various cell types in adjuvant-induced protective immune responses is vital for an effective vaccine design. We have investigated the role of cell-specific MyD88 signaling in vaccine adjuvant activity *in vivo*, using Neisserial porin B (PorB), a TLR2 ligand-based adjuvant, compared with an endosomal TLR9 ligand (CpG) and toll-like receptor (TLR)-independent (alum, MF59) adjuvants. We found that intact MyD88 signaling is essential, separately, in all three antigen-presenting cell types [B cells, macrophages, and dendritic cells (DCs)] for optimal TLR ligand-based adjuvant activity. The role of MyD88 signaling in B cell and DC in vaccine adjuvant has been previously investigated. In this study, we now demonstrate that the immune response was also reduced in mice with macrophage-specific MyD88 deletion (Mac-MyD88^−/−^). We demonstrate that TLR-dependent adjuvants are potent inducers of germinal center (GC) responses, but GCs are nearly absent in Mac-MyD88^−/−^ mice following immunization with TLR-dependent adjuvants PorB or CpG, but not with TLR-independent adjuvants MF59 or alum. Our findings reveal a unique and here-to-for unrecognized importance of intact MyD88 signaling in macrophages, to allow for a robust vaccine-induced immune responses when TLR ligand-based adjuvants are used.

## Introduction

Immune adjuvants are necessary in most, if not all, vaccines, to enhance efficacy by increasing the immunogenicity of poorly immunogenic antigens. Adjuvants can be either exogenously added or are inherent in the vaccine preparations, e.g., live attenuated or killed whole organism vaccines. The majority of adjuvants originate from microbial components, referred to as pathogen-associated molecular patterns (PAMPs). Our immune system has evolved to respond to these PAMPs and stimulate innate immune responses through germline-encoded pattern recognition receptors. Toll-like receptors (TLRs) are some of the best-studied PPRs used by the immune system to respond to PAMPs. Vaccines containing TLR ligand-based adjuvants activate and trigger innate immune responses to enhance a potential protective immune response induced by vaccines (1).

Adjuvants shape the character and intensity of responses triggered *in vivo*. Moreover, the mechanism of action for most adjuvants is not very well understood. Therefore, in this study, a variety of well-characterized TLR-dependent [porin B (PorB)—TLR2, CpG—TLR9] and TLR-independent adjuvants (alum, MF59) were examined. PorB is the major outer membrane protein from *Neisseria meningitidis* (2). This protein has been investigated as a potential anti-Neisserial vaccine candidate (3–5), through this work it was found that this protein had potent immune stimulatory abilities beyond its own potential use as a vaccine. PorB stimulates antigen-presenting cells (APCs) through direct interaction with TLR2/TLR1 heterodimers and requires the adaptor protein MyD88 for this stimulation to occur (6–9). In this study, we compared PorB with an endosomal TLR9-dependent adjuvant; CpG DNA (CpG). CpG is a TLR9/MyD88-dependent adjuvant composed of unmethylated bacterial DNA motifs (10). In addition, PorB was also compared to alum salt and MF59, two TLR-independent adjuvants. Alum is the first adjuvant approved by the FDA, has been in use for over 80 years in millions of vaccine doses, and induces mainly Th2 type immune responses and its effect is MyD88 and TRIF independent (11). MF59, consists of an oil-in-water squalene solution, has been a licensed adjuvant in the formulation of the influenza vaccines since 1994 in Europe (Novartis Vaccines). The adjuvanticity of MF59 is still being investigated and appears to be MyD88 dependent but TLR independent (12).

Toll-like receptors are commonly expressed in various cell types, especially APCs, including dendritic cells (DCs), B cells, and macrophages (13, 14). TLR ligand-based adjuvants are used in vaccines to stimulate these immune cells, to induce, and to boost protective immune responses, linking the innate and adaptive immunity (1). Investigators have shown that incorporation of glucopyranosyl lipid adjuvant (binding to TLR4) in an oil-/water-emulsified malaria vaccine or in an investigative tuberculosis vaccine can greatly increase diversity of the antibody repertoire and enhance protection in animal models (15, 16). The successful yellow fever vaccine has been shown to activate multiple subsets of DCs by signaling through a specific set of TLRs (TLR2, 7, 8, and 9) (17). Absence of one of these TLRs altered and diminished the type of responses by the vaccine *in vitro* in DCs (17).

Previous studies have established that TLR recognition and signaling via the MyD88 adaptor protein are crucial for immune responses triggered by TLR ligand-based vaccine formulations (18). MyD88 signaling in B cells *in vivo* is critical for induction of antibody-secreting cells upon vaccination (19). It is also important for induction of antiretroviral germinal center (GC) response (20) and required for induction of functional CD4 T cells producing IFN-γ for the control of IgG2c subclass production (21). Generation of T cell-dependent (TD) antigen-specific antibody responses requires TLR and MyD88 signaling in naïve human B cells and that TLR stimulation of DCs alone is not sufficient to induce TD B-cell responses (22, 23). GC formation is crucial for the production of antibody-secreting cells including memory B cells, which produce class switched isotypes and high affinity antigen-specific antibodies, and is essential for a rapid recall response to antigens (24). Intact TLR-MyD88 signaling in B cells and DCs has previously been shown to be important for induction of robust GCs and antibody production upon stimulation with TLR ligand-based adjuvants (25). Intrinsic DC-MyD88 signaling *in vivo* was essential to trigger Th2/Th1 cells (26), the induction of a robust humoral response with CpG as a TLR9 ligand (27) and activation of adaptive immune responses (28). However, the contribution of macrophage intrinsic MyD88 signaling in TLR ligand-based vaccine adjuvant responses has not been investigated.

The impact of *in vivo* MyD88 signaling in specific immune cells, e.g., B cells, DCs, or macrophages, on TLR ligand-based immunomodulation, and subsequent vaccine efficacy was examined in this study. We used the loxP/cre recombinase system to conditionally knock out MyD88 in individual APC types *in vivo*. Immunizations of these mice revealed the importance of functional cell-specific MyD88 signaling in the adjuvanticity of TLR-dependent and -independent adjuvants. Our findings confirm the importance of intact *in vivo* MyD88 signaling in B cells and DCs and reveal a here-to-for unrecognized importance in macrophages, demonstrating its contribution to a robust vaccine-induced immune response, including GC formation, when TLR ligands are used as adjuvants.

## Animals and Methods

### Animals

All mice including wild-type (WT) C57BL/6J were purchased from Jackson Laboratories. MyD88^flox/flox^ (MyD88^tm1Defr^, stock # 008888) (28) mice were bred with CD19 Cre (Cd19^tm(cre)Cgn^, stock # 006785) (29), CD11c Cre [Tg(Itgax-cre)1-1Reiz, stock # 008068] (30) or Lysm Cre (Lyz2^tm1(cre)Ifo^, stock # 004781) (31) mice then crossed again with MyD88^flox/flox^ to generate MyD88^flox/flox^ homozygous and heterozygous Cre mice. MyD88^flox/flox^ CD19Cre mice have a MyD88 deletion in CD19^+^CD3^−^ B cells (B-Cell-MyD88^−/−^). MyD88^flox/flox^ CD11cCre mice have MyD88 deleted in CD11c^+^CD11b^−^ DCs (DC-MyD88^−/−^ mice). MyD88^flox/flox^ LysmCre mice have a MyD88 deletion in CD11b^+^F4/80^+^ macrophages (Mac-MyD88^−/−^ mice). All mice were maintained in the Association for Assessment and Accreditation of Laboratory Animal Care International accredited facility at Boston University School of Medicine Laboratory Animal Science Center. All animal experiments were conducted under the approved Institutional Animal Care and Use Committee (IACUC) protocol for Dr. Wetzler’s Laboratory at Boston University. Mice were genotyped by PCR using primer sequences recommended by Jackson Laboratories.

### Immunizations

Wild-type, B-cell-MyD88^−/−^, DC-MyD88^−/−^, and Mac-MyD88^−/−^ mice between the ages of 6 and 12 weeks were immunized subcutaneously three times at 2-week intervals. Mice were immunized with 10 μg of *N. meningitidis* PorB, or CpG (ODN1826, Invivogen) admixed with 10 μg of ovalbumin (OVA). Alum vaccinations contained 200 μg of aluminum hydroxide, alum (Sigma) mixed with 10 μg of OVA. MF59-based vaccines were prepared by mixing 50 μl of MF59 (gift from Anja Seubert, Novartis Vaccines and Diagnostics) with 50 μl PBS containing 10 μg of OVA. Mice were immunized with either PBS or 10 μg OVA protein as controls. There were at least four mice per group and single experiments were repeated to obtain a total of eight mice per group. PorB was purified from *N. meningitidis* strain H44/76 Δ-1/4 (32) using protein extraction and column chromatography as previously described (33). Activity of PorB and endotoxin content were examined by stimulation of WT, MyD88^−/−^, TLR2^−/−^, and TLR4^−/−^ BMDM, silver staining, and LAL assay (Pierce Endotoxin Kit from Life Technologies), no endotoxin was found in any preparation. The amount of adjuvants and antigen utilized were based on previously published studies and falls within the best range of their efficacy (6, 34–36). Sera were collected via tail bleed prior to each immunization and 2 weeks after the last immunization. For serum cytokine measurements, preimmune sera as well as sera from 4 h post immunization were collected. This time point was selected based on our examinations of different time points and previously published work (37).

### Measurement of Antigen-Specific Antibodies

Sera were assayed for OVA-specific immunoglobulins by enzyme-linked immunosorbent assay as previously described (38, 39). Briefly, wells were coated with OVA (5 μg/mL) in carbonate buffer and incubated overnight at 4°C. Sera were sequentially diluted starting at 1:50 and added to the previously coated wells, and incubated overnight at 4°C. Alkaline phosphatase-conjugated anti-mouse total IgG or IgM (Sigma Aldrich, St. Louis, MO, USA) were added and the plates developed with one-step *p*-nitrophenyl phosphate (Pierce, Rockford, IL, USA) and the optical density at 405 nm was measured on a SpectraMax190 Microplate Reader (Molecular Devices, Sunnyvale, CA, USA). Colorimetric values were converted to nanograms/milliliter, according to standard curves generated by known amounts of IgG as previously described (32) or known amounts of IgM using GraphPad Prism.

### Sectioning for Fluorescent Microscopy

Spleens were obtained 7 days after the second immunization of the mice and were embedded in optimal cutting temperature medium (Richard Allan Scientific, Kalamazoo, MI, USA) in molds (ThermoFisher) and flash frozen in an isopropanol dry ice bath and stored at −80°C. Sectioning was performed on a cryostat (Microm HM 550, Microm International GmbH, Walldorf, Germany). Embedded spleens were removed from molds, placed on mounts, and allowed to equilibrate to the −20°C internal temperature of the cryostat for about 15 min. For initial trimming 20 μm slices were made until the tissue was visible at the surface of the block. Sections of 8 μm thickness were obtained and placed on lysine treated slides (Colorfrost Plus, ThermoFisher). Six sections were cut per mouse and a total of 18 sections for the three mice per immunization group. About 24 μm or more of tissue were cut between the collected sections and special attention was given to the location of the GC upon imaging to prevent quantitation of the same GCs.

### Confocal Microscopy

Sections were air dried for 15 min at room temperature, then fixed in acetone at −20°C for 10 min and afterward air dried for 10 min. Sections were rehydrated in tris-buffered saline solution (TBS) with 0.05% Tween-20 (TBS-T) then blocked for 20 min at room temperature with TBS-T with 5% BSA. Sections were rinsed with TBS-T and then stained with antibodies for 1 h followed by two rinses with TBS-T and incubated in a TBS-T bath for 5 min on an orbital shaker. The following antibodies were used at 1 to 200 dilution: FITC Rat anti-Mouse B and T cell Activation Antigen Clone: GL7 (Beckton Dickinson Biosciences, San Jose, CA, USA), PE anti-Mouse IgD Clone: 11-26c.2a (BioLegend, San Diego, CA, USA) and Alexa Fluor 647 anti-Mouse TCR-β Clone: H57-597 (BioLegend, San Diego, CA, USA). Stained sections were mounted in fluoroshield mounting medium with DAPI (Abcam, Cambridge, MA, USA), covered with a cover glass, and dried overnight. A Leica TCS Sp5 DMI6000 confocal microscope (Leica AG, Wetzlar, Germany) was used to examine the sections using the Leica Application Suite Advanced Fluorescence software. Four separate lasers were used to excite the four different fluorochromes and the appropriate gating was applied to prevent emission spectral overlap during capture. The 10× objective (HC PL FLUOTAR 10.0× 0.3 Dry) with a numerical aperture of 0.3 was used to capture the images with four lines average at 200 Hz with a resolution of 1,024 × 1,024 and at 16 bit. The images were analyzed and area measured using Image processing analysis in Java (ImageJ, NIH). Areas less than 400 μm^2^ were excluded, as they could not be measured accurately using the freehand selection feature in the software.

### Flow Cytometry

Germinal center cells were examined using a BD LSRII Flow Cytometer (Beckton Dickinson Biosciences, San Jose, CA, USA) in the Boston University Flow Core. Spleens from mice (*N* = 3) were harvested 7 days after the second immunization and single cells suspension were prepared. Red Blood Cells were lysed with ammonium-chloride-potassium lysis buffer prepared in house (ACK Lysis Buffer). Cells were washed with cold FACS buffer (0.5% FBS + 2 mM EDTA in PBS) then stained for 30 min at 4°C with fluorescently labeled antibodies in 40 μl of FACS buffer in 96-well plate then washed and transferred in FACS cluster tube for analysis. The following antibodies were used: FITC Rat anti-Mouse B and T cell Activation Antigen Clone: GL7, APC Rat anti-Mouse CD4 Clone RM4-5, Biotin Rat anti-Mouse CD45R/B220 Clone: RA3-6B2 (Beckton Dickinson Biosciences, San Jose, CA, USA), and PE/Cy7 anti-Mouse CD19 Clone: 6D5 and PE Strepavidin (BioLegend, San Diego, CA, USA). Forward and side scatter gates were used to identify live cells (gating strategies were similar to Figures S1A–D in Supplementary Material) and cells that were CD4^−^, B220^+^, CD19^+^, and GL7^+^ were gated as GC B cells. All gates were based on PBS immunized mice as negative control. For lymphoid organs composition: single-cell suspensions from spleens and inguinal, axillary and brachial lymph nodes from WT, B-Cell-MyD88^−/−^, DC-MyD88^−/−^, and Mac-MyD88^−/−^ mice were prepared as described above. Cells were stained with fluorescently labeled antibodies and the number of CD8^+^ T-cells, CD11b^+^F4/80^+^ Macrophages, CD19^+^ B cells, CD11c int B220^+^ Ly6C^+^ CD19^−^ pDCs, CD11c^+^B220^−^ DCs, CD11b^+^Ly6G^+^ PMNs, NK1.1^+^CD4^−^ T-cells, and CD4^+^ T-cells were measured by flow cytomteric analysis. Two mice per genotype were examined. Differences between the number of cells in WT and the different MyD88 Floxed mice were calculated using one-way ANOVA with Bonferroni test. Analysis was performed using FlowJo (Tree Star, Ashland, OR, USA).

### Measurements of Cytokines

Cytokine levels were measured from sera obtained 4 h post each immunization and compared to preimmune sera. Individual mouse sera (*N* = 4 mice per immunization group) screened on a MAGPIX XMAP instrument (Luminex, Austin, TX, USA) in duplicates using Mouse 20-plex cytokines kits (Life Technologies). Individual standard curves were generated for each cytokines and analyzed using the Luminex Xponent software. This experiment was performed twice. Unknown sample concentration was extrapolated from standard curves for single analytes. All values outside of the standard curve limit were rejected.

### Statistical Analysis

Statistics were calculated using with GraphPad Prism 6.0. Differences in OVA-IgG concentration between WT and the different MyD88 Floxed mice were calculated using one-way ANOVA with Tukey’s test. Differences in percentage of GC cells and area of GCs were calculated using the non-parametric Mann–Whitney *U* test. In all experiments, significance levels were defined as ns = *P* > 0.05, *P < 0.05, **P < 0.01, ***P < 0.001, and ****P < 0.0001.

## Results

### The Requirement of *In Vivo* MyD88 Signaling in Macrophages, B Cells, and DCs, Individually, for TLR Ligand-Based Adjuvant Function

We have previously demonstrated that PorB lacks adjuvant activity in MyD88 KO mice (9). In this study, we examined the effect of deficient MyD88 signaling, separately, in each APC type, in regard to the adjuvant activity of PorB and CpG as TLR2 and TLR9 ligand-based adjuvants, respectively. We used MyD88 conditional KO mice, with APC-specific MyD88 deletion in B cells, DCs, or macrophages. MyD88^flx/flx^CD19Cre, MyD88^flx/flx^CD11cCre, or MyD88^flx/flx^LysmCre mice (termed B-cell-MyD88^−/−^, DC-MyD88^−/−^, and Mac-MyD88^−/−^ mice) were generated by breeding MyD88^flx/flx^ mice with mice expressing Cre recombinase under cell-specific promoters. Previous studies have shown efficient deletion of MyD88 in DCs and B cells from the DC-MyD88^−/−^ mice and B-cell-MyD88^−/−^ mice, respectively (28), and macrophages from the Mac-MyD88^−/−^ mice (40) utilizing this method. CD3^−^CD11c^+^CD11b^−^ DCs, CD3^−^CD19^+^ (B cells), and CD11b^+^F4/80^+^ (macrophages) were sorted from WT, DC-MyD88^−/−^, B-cell-MyD88^−/−^, and Mac-MyD88^−/−^ mice as shown in Figure S1 in Supplementary Material. MyD88 was efficiently deleted in the different mouse strains as measured by quantitative real-time PCR (Figure S2 in Supplementary Material) performed on genomic DNA isolated from sorted cells (Figure S1 in Supplementary Material). Deletion of MyD88 was confined to a specific cell type while the other APC types expressed normal levels of MyD88 (Figure S2 in Supplementary Material). Percentages and numbers of CD8^+^ T-cells, CD11b^+^F4/80^+^ Macrophages, CD19^+^ B cells, CD11c int B220^+^ Ly6C^+^ CD19^−^ plasmacytoid DCs, CD11c^+^B220^−^ DCs, CD11b^+^Ly6G^+^ PMNs, NK1.1^+^CD4^−^ T-cells, and CD4^+^ T-cells from spleen and six different lymph nodes in these three transgenic mice were also examined. There were no significant differences in the major cell types as compared to WT mice suggesting that deletion of MyD88 in the specific cell types did not affect these cell populations in the lymphoid organs (Figures S3 and S4 in Supplementary Material).

**Figure 1:**
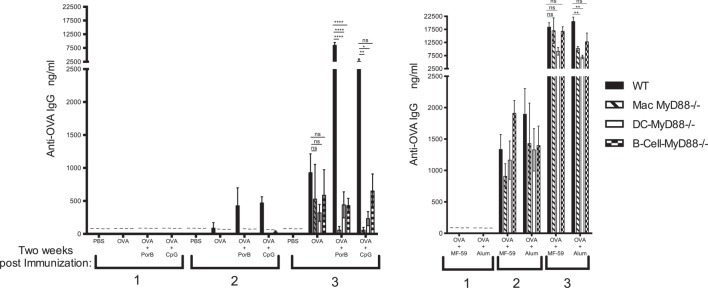
**Intact *in vivo* MyD88 signaling in B cells, dendritic cells (DCs), or macrophages is required for robust vaccine-induced humoral responses by toll-like receptor (TLR)-based adjuvants**. Total ovalbumin (OVA)-IgG concentrations were measured by enzyme-linked immunosorbent assay (ELISA) following immunization with **(A)** PBS, OVA alone or mixed with TLR-dependent adjuvants PorB, CpG or **(B)** with TLR-independent adjuvant MF59 or alum. Wild-type, Mac-MyD88^−/−^, DC-MyD88^−/−^, and B-cell-MyD88^−/−^ mice, respectively, were immunized three times at 2-week interval. The results shown are from samples collected 2 weeks after each immunization and representative of two experiments with a total of four to eight mice per immunization. Statistics were calculated based on a non-parametrical one-way ANOVA with Tukey’s test (ns, not significant *P* > 0.05, *P < 0.05, **P < 0.01, ***P < 0.001, and ****P < 0.0001). Symbol (–) indicates that IgG levels were below detectable level.

Wild-type, B-cell-MyD88^−/−^, DC-MyD88^−/−^, and Mac-MyD88^−/−^ mice were immunized with OVA antigen alone or admixed with TLR-dependent or -independent adjuvants. The vaccine-induced antibody responses were measured 2 weeks after each immunization (Figure 1). OVA-specific IgM was only detected in mice immunized with OVA alone and only 14 days after the second immunization, for all examined immunization groups and time points (data not shown). One vaccination was not sufficient to trigger an OVA-specific IgG response in either TLR-dependent or -independent adjuvanted vaccines (Figure 1). Two weeks after the second vaccination WT mice had a low but detectable antigen-specific response to OVA when the TLR-dependent and -independent adjuvants were utilized, but these differences were not significantly different from the MyD88 conditional KO mice when PorB, MF59, or alum were used as adjuvants (Figure 1). Notably, OVA/CpG immunized WT mice had significantly higher OVA-specific IgG after the second immunization when compared to the various MyD88 conditional KO mice (Figure 1).

Wild-type mice mounted a robust anti-OVA antibody response after three immunizations using any of the adjuvant formulations, while immunizations of the conditional MyD88 KO mice revealed that intact signaling through MyD88 was required in B cells, macrophages, or DCs for optimal adjuvant activity of the TLR2-dependent adjuvant PorB (Figure 1A). Similar results were observed when CpG, an endosomal TLR9 ligand, was used as an adjuvant. This indicates that intact MyD88 signaling is crucial for either TLR2 or TLR9 ligand-based adjuvants, which rely on the MyD88 adaptor protein to induce resilient antibody responses (Figure 1A). TLR-independent adjuvants in contrast, e.g., MF59 or alum, were able to trigger a vigorous humoral response against OVA in mice lacking MyD88 in any of the three APC types (Figure 1B). This suggests that the decrease in the humoral response when PorB or CpG were used as adjuvants is not due to an intrinsic defect in these mice, but, rather, to the specific absence of MyD88 signaling in all these APC types. These results demonstrate the importance of MyD88 signaling in all three APC types when TLR ligand-based adjuvants are used. In regard to macrophages, this is the first time to our knowledge that MyD88 signaling was shown to be as important in this process.

### *In Vivo* Cytokine Responses Require Intact MyD88 Signaling in B Cells, DCs, and Macrophages

**Figure 2:**
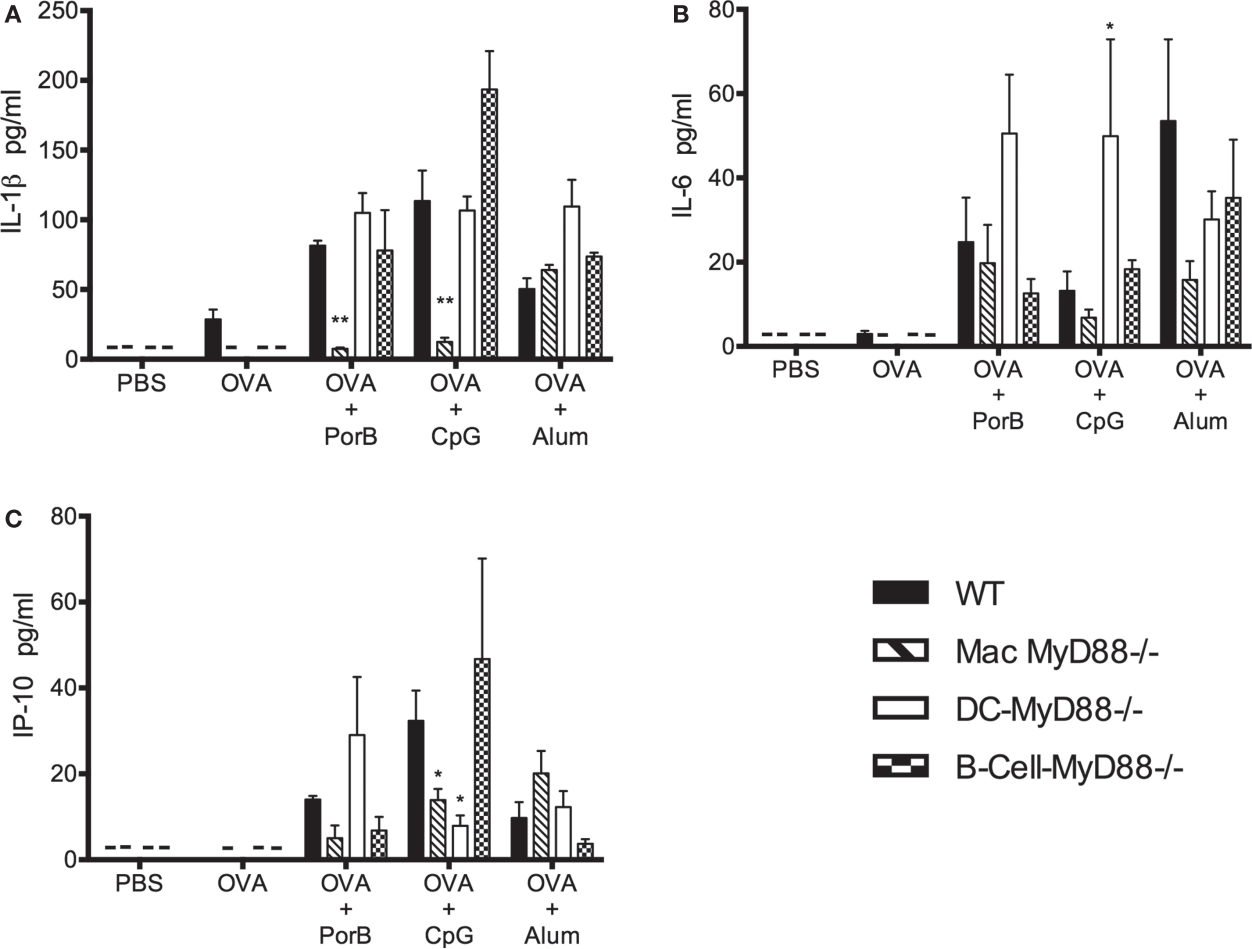
**Effect of MyD88 signaling on cytokine induction upon vaccination**. *In vivo* MyD88 signaling is important for the induction of **(A)** IL-1β, **(B)** IL-6, and **(C)** IP-10 cytokines. Serum cytokine levels were determined in vaccinated wild-type (WT), Mac-MyD88^−/−^, B-cell-MyD88^−/−^, and DC-MyD88^−/−^ mice immunized two times at 2 weeks interval and serum collected 4 h after of the second immunization with PBS, ovalbumin (OVA), OVA + PorB, OVA + CpG, or OVA + alum measured by Luminex magnetic bead-based multiplex assay. The results shown are representative of one experiment (*n* = 4 mice); serum was analyzed individually in duplicates. This experiment was repeated twice. Symbol (–) indicates that cytokine levels were below detectable level. One-way ANOVA with Tukey’s test were used to calculate statistical differences between experimental groups and treatments (ns, not significant *P* > 0.05, *P < 0.05, **P < 0.01). All comparisons were made to WT mice.

**Figure 3:**
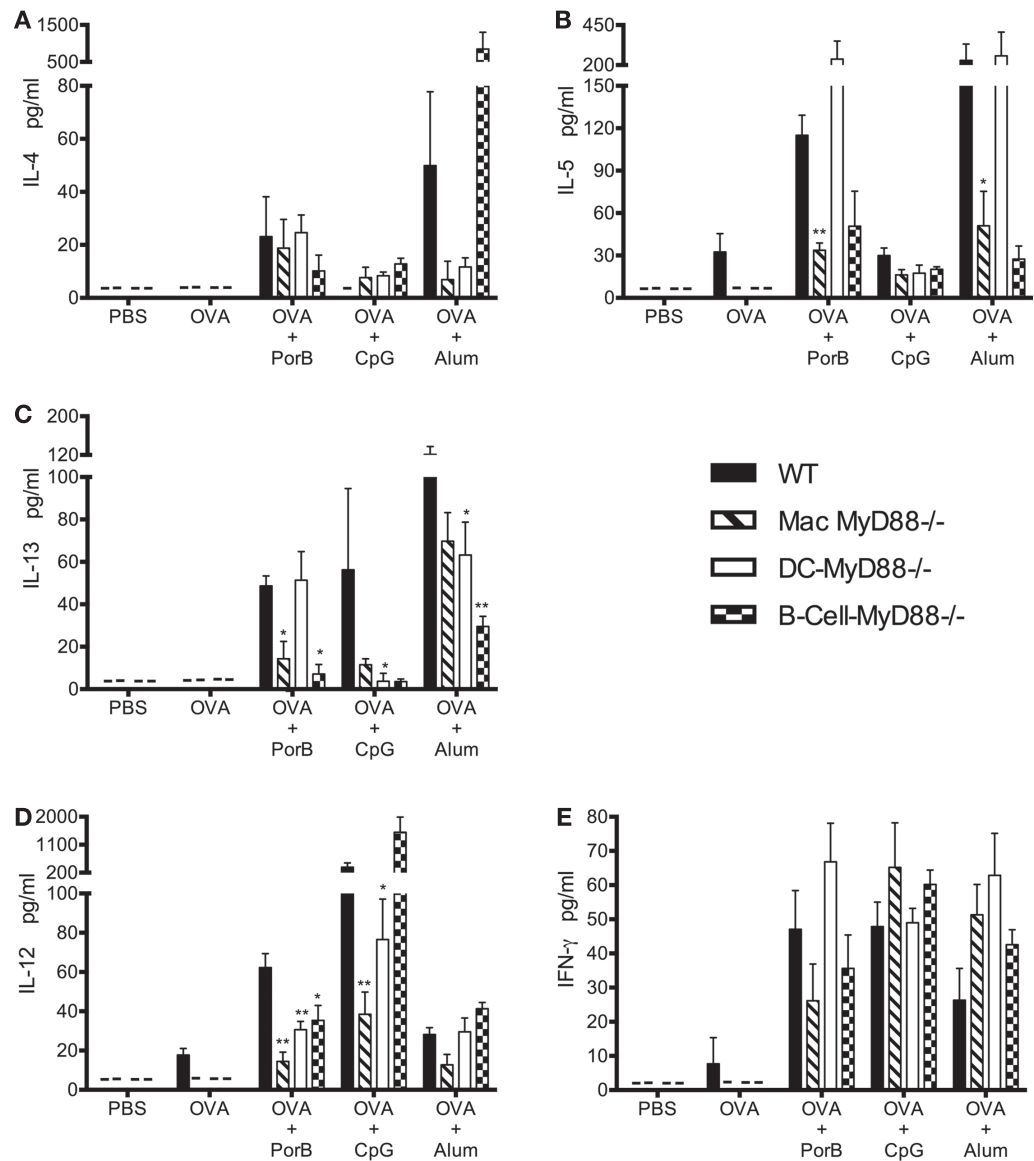
***In vivo* MyD88 signaling in macrophages differentially affects the induction of Th2 and Th1 type cytokines upon immunization with toll-like receptor ligand-based adjuvants**. **(A–C)** Th2 type (IL-4, IL-5, and IL-13) and **(D,E)** Th1 type (IL-12 and IFN-γ) cytokines level in individual mouse serum of wild-type (WT), Mac-MyD88^−/−^, B-cell-MyD88^−/−^, and DC-MyD88^−/−^ mice immunized two times at 2 weeks interval and serum collected 4 h after of the second immunization with PBS, ovalbumin (OVA), OVA + PorB, OVA + CpG, or OVA + alum measured by Luminex magnetic bead-based multiplex assay. The results shown are representative of one experiment (*n* = 4 mice); serum was analyzed individually in duplicates. This experiment was repeated twice. Symbol (–) indicates that cytokine levels were below detectable level. One-way ANOVA with Tukey’s test were used to calculate statistical differences between experimental groups and treatments (ns, not significant *P* > 0.05, *P < 0.05, **P < 0.01). All comparisons were made to WT mice.

Adjuvanted subunit vaccines almost always require repeated booster injections to induce sufficient protective IgG levels. The cytokine production during the initial induction of vaccine-associated immune responses is important for the quality, robustness, and longevity of the response. Therefore, we measured IL-1β, IL-6, IP-10, and the Th2/Th1 cytokines from sera collected 4 h after each immunization (Figures 2 and 3). The selected cytokines were chosen because their essential roles in vaccine-induced humoral and cellular immune responses (41–46). WT, DC-MyD88^−/−^, B-cell-MyD88^−/−^, and Mac-MyD88^−/−^ mice were immunized with OVA admixed with TLR-dependent adjuvants PorB, CpG, or the TLR-independent adjuvant alum. Serum cytokine levels were undetectable or very low 4 h after the first immunization for most of the Th2/Th1 cytokines (data not shown). However, 4 h after the second immunization cytokines levels were increased in WT mice immunized with adjuvanted vaccines. Moreover, OVA/PorB immunized WT mice developed high levels of IL-1β, IL-6, and IP-10 (Figures 2A–C), while OVA/PorB immunized Mac-MyD88^−/−^ mice had similar levels of IL-6 but diminished levels of IL-1β and IP-10 when compared to the WT mice (Figures 2A–C). In addition, deletion of MyD88 in macrophages resulted in a more profound decrease in IL-1β, and IP-10 cytokines when TLR ligand-based adjuvants were used compared to TLR-independent adjuvants (Figures 2A–C). For example, IL-1β and IP-10 levels in mice immunized with OVA/alum were not decreased in the Mac-MyD88^−/−^ mice as compared to WT mice.

Interestingly, in contrast to the Mac-MyD88^−/−^ mice, IL-6 levels were increased in the DC-MyD88^−/−^ mice compared to WT mice when immunized with either OVA/PorB or OVA/alum, suggesting that the DCs may have some control over IL-6 induction that is MyD88 dependent. IP-10 and IL-6 levels were decreased in OVA/PorB immunized B-cell-MyD88^−/−^ mice when compared to WT mice. In contrast to Mac-MyD88^−/−^ or DC-MyD88^−/−^ mice, IL-1β levels from B-cell-MyD88^−/−^ mice remained comparable to WT mice.

Th1 and Th2 type cytokines in sera of vaccinated mice were measured four hours after each immunization. Following the first immunization there were very low or undetectable levels of Th2/Th1 type cytokines. However, four hours after the second immunization, formulations containing PorB induced high levels of IL-4, IL-5, and IL-13 (Th2 type cytokines, Figures 3A–C) as well as IFN-γ and IL-12 (Th1 type cytokines, Figures 3D,E) in WT mice. Selective deletion of MyD88 in macrophages resulted in a significant decrease in most of these cytokines except for IL-4 and IFN-γ, which remained unchanged (Figures 3A,D). As expected, TLR-dependent adjuvant CpG induced mainly a Th1 type response as determined by cytokine responses (Figure 3) (10). Lack of MyD88 signaling in macrophages *in vivo* resulted in a significant decrease in IL-12, but not IFN-γ, when CpG was used as an adjuvant (Figures 3D,E), similar to the results observed with PorB. The TLR-independent adjuvant alum mainly induced Th2 type cytokines (as expected) in WT mice (47), and deletion of MyD88 in macrophages, *in vivo*, resulted in non-significant decreases in the levels of the Th2 type cytokines except for IL-5, which was significantly decreased (Figures 3A–C). Interestingly, IL-4 levels were also decreased when alum was used as a vaccine adjuvant in Mac-MyD88^−/−^ mice. This is in contrast to the TLR2 ligand-based adjuvant, PorB. Th2/Th1 type cytokine responses were differentially affected with the lack of MyD88 signaling in B cells or DCs *in vivo*. For example, DC-MyD88^−/−^ mice immunized with vaccine containing TLR2-dependent adjuvant PorB had a high level of systemic Th2 type cytokines, unlike what was observed with Mac-MyD88^−/−^ mice. A different Th1 type cytokine profile was also observed in DC-MyD88^−/−^ and B-cell-MyD88^−/−^ mice upon immunization with TLR ligand-based adjuvants. The heterogeneity in the different adjuvant responses is likely due to the varied mechanism by which these adjuvants induce their responses.

### Intact *In Vivo* MyD88 Signaling in Macrophages Is Required for GC Formation Induced by TLR Ligand-Based Adjuvants

The anti-OVA immune response induced by OVA/PorB or OVA/CpG in Mac-MyD88^−/−^ mice was much less as compared to WT mice (Figure 1A). The most effective humoral immune responses induced by vaccination rely on a class switch recombination, antibody somatic hypermutation, and affinity maturation, which all occur in GCs. A unique subset of macrophages resides in and around the lymphoid follicles where GCs form, MARCO1^+^ marginal zone macrophages (MZM) or CD169^+^ metallophilic macrophages. Together, along with other subcapsular cell types, these macrophages orchestrate and modulate the resulting humoral immune response. We hypothesized that the function of these subcapsular macrophages cannot be recapitulated by DCs or B cells, and therefore, the lack of MyD88 signaling in these cells could explain the significant effect on the OVA-specific IgG response in the Mac-MyD88^−/−^ mice. We do not believe that the antigen presentation by macrophages, which could be affected by a defect in MyD88 signaling in these cells, because DC function in the Mac-MyD88^−/−^ mice should be normal, including its major antigen presentation function, but still, normal DCs cannot substitute for the function of macrophages lacking MyD88. Therefore, we next investigated the effect of MyD88 deficiency on the induction of GCs upon immunization with OVA formulated with PorB, CpG, or alum.

**Figure 4:**
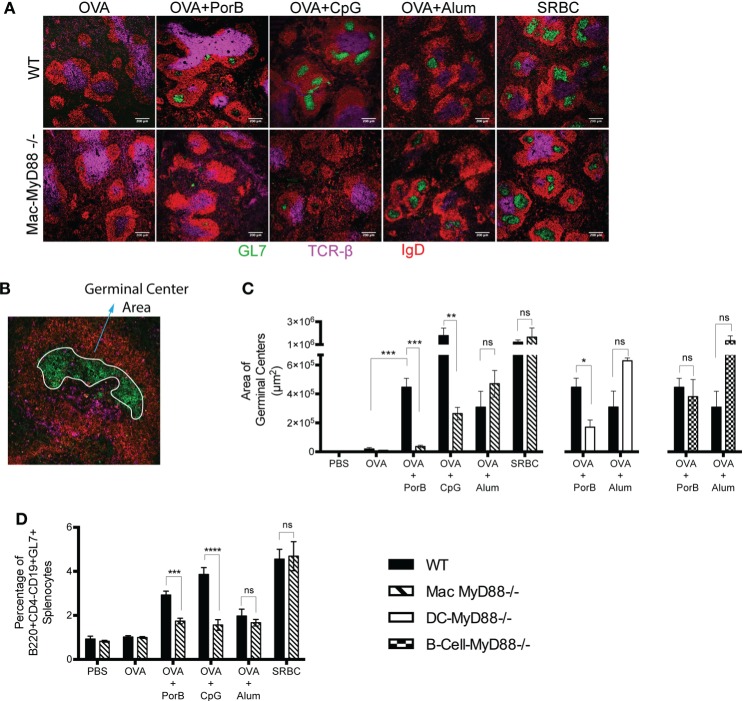
**Formations of germinal centers (GCs) in the spleen are dependent on *in vivo* MyD88 signaling in macrophages upon immunization with toll-like receptor ligand-based adjuvants**. **(A)** GC responses examined by confocal microscopy 7 days after the second immunization, 8 μm thick spleen sections were prepared and examined as described “Animals and Methods.” Sections were stained for IgD follicular B cell marker (red), GC B cell marker GL7 (green), and T cell marker TCR-β (cyan). **(B)** A spleen section with the GC area measured using the freehand selection tool in ImageJ, here the area of GC (green) is encircled in white. **(C)** Quantitation of GC areas by ImageJ. There were three mice per immunization group, 6 sections per spleen that are at least 40 μm apart and a total of 18 sections were examined per group. **(D)** Percentage of GC cells from spleen was measured by flow cytometry 7 days after the second immunization and gating for B220^+^CD4^−^CD19^+^GL7^+^GC cells. The results shown are representative of at least two experiments with a total of eight mice per immunization group for the flow cytometry analysis experiment. One-way ANOVA with Tukey’s test were used (ns, not significant *P* > 0.05, *P < 0.05, **P < 0.01, ***P < 0.001, and ****P < 0.0001).

Splenic GC size and number were determined at various time points after immunizations. Seven days after the second immunization yielded the most consistent and replicable production of GCs in WT mice and was consequently used to assess GC responses for all further experiments. We found a significant decrease in the area of GC in the spleens of Mac-MyD88^−/−^ mice when compared with WT mice, after immunization with TLR ligand-based adjuvant PorB or CpG (Figures 4A–C). Interestingly, Mac-MyD88^−/−^ mice form equivalent GC areas, as compared to WT mice, when immunized with TLR-independent adjuvant alum (similar to the antibody responses) or when injected with sheep red blood cells as a positive control (Figures 4A,C). This demonstrates that the decrease in GC formation when TLR ligand-based adjuvants were used was not due to an intrinsic defect in GC formation in the Mac-MyD88^−/−^ mice, but rather due to the specific lack of signaling through MyD88.

As mentioned, we observed a decrease in OVA-specific IgG in DC-MyD88^−/−^ and B-cell-MyD88^−/−^ mice, when immunized with TLR ligand-based adjuvants (Figure 1). This is consistent with previous studies demonstrating that TLR9 signaling in DCs and B cells controls the magnitude of the GC reaction upon immunization with vaccine formulated with the TLR9 ligand, CpG (48). Therefore, we compared GC formation in Mac-MyD88^−/−^ mice, with DC-MyD88^−/−^ and B-cell-MyD88^−/−^ mice. We found a similar decrease in GC formation in DC-MyD88^−/−^ mice in comparison with Mac-MyD88^−/−^ mice, when immunized with OVA formulated with PorB as adjuvant. GC formation was not impaired DC-MyD88^−/−^ mice immunized with OVA along with TLR-independent adjuvant alum. We did not observe a decrease in GC formation in B-cell MyD88^−/−^ mice immunized with either OVA/PorB (Figure 4C), although we did find that these mice did not mount an anti-OVA humoral response (Figure 1). This suggests that intact *in vivo* MyD88 signaling in B cells is not required for GC formation, but is required for specific antibody production when PorB is used as an adjuvant. This disconnection of GC formation vs. antibody production in the B cell MyD88^−/−^ mice is unique and suggests that MyD88 in B cells is essential for TLR-mediated induction plasma cells and antibody production but not for GC formation. Further studies are planned to investigate this phenomenon.

Ovalbumin formulated with CpG induced large GC areas as compared to any other adjuvants in WT mice (Figure 4C) and triggered the highest percentage of CD4^−^B220^+^CD19^+^GL7^+^GC B cells (GC B cells) (Figure 4D). GCs were decreased in Mac-MyD88^−/−^ immunized with OVA^+^CpG (Figure 4D). However, many of the small GC areas triggered by CpG in Mac-MyD88^−/−^ mice were larger than 400 μm^2^ and therefore measurable by ImageJ in contrast to PorB formulation, which yielded mainly GC areas smaller than 400 μm^2^. This may explain why there was a residual amount of GC area detected in Mac-MyD88^−/−^ mice for CpG formulated vaccines (Figure 4C). Nevertheless, we found a significant decrease in GC formation with TLR ligand-based adjuvant in Mac-MyD88^−/−^ mice in both analysis methods, quantification of immunofluorescence, and flow cytometry. Mice immunized with OVA alone in contrast produced very few GL7^+^ areas, and PBS-injected mice showed none by confocal microscopy. Flow cytometric analysis allowed a more precise survey of the entire spleen with an accurate assessment of the presence GC B cells (B220^+^CD4^−^CD19^+^GL7^+^). This presumably accounts for the limitation of missing the formation of GC in immunofluorescence quantification and their detection in the flow cytometric analysis. The residual GC cells in non-immunized mice are likely due to baseline responses to other antigens that the mice are exposed to in the animal facility and/or background staining of the cells. Notably, there was no decrease in GC area and cell numbers with vaccines containing TLR-independent adjuvants in these Mac-MyD88^−/−^ mice (Figure 4D). These findings demonstrate a major role of intact MyD88 signaling in macrophages for robust GC formation (along with antibody formation, as previously described) when TLR ligands are used as adjuvants.

## Discussion

In the present study, we provide detailed insight into the *in vivo* role of TLR signaling through MyD88 in B cells, DCs, or macrophages for TLR ligand-based vaccine adjuvant activity. MyD88 is an essential adaptor-signaling molecule for all TLRs except TLR3 and the TRIF/TRAM signaling pathway for TLR4 (49). Complete MyD88^−/−^ mice have reported defects in the induction of humoral responses using OVA and LPS in Complete Freund’s adjuvant (22), live virus infections (50, 51), with inactivated influenza virus (51, 52), or with OVA/PorB as we have previously demonstrated (39). We found that *in vivo* MyD88 signaling, individually, in three different APC types (B cells, macrophages, or DCs), was essential for the adjuvant activity of PorB and CpG, both TLR ligands that signal through MyD88. The most unique finding of this study was that mice with a macrophage-specific MyD88 deletion had a decrease in T-cell-dependent antibody responses upon immunization with vaccines formulated with TLR ligand-based adjuvants PorB or CpG, as compared to immunized WT mice. In contrast, TLR-independent adjuvants, alum and MF59, were able to induce a robust antibody response in mice with MyD88 deletion in specific APC types.

Previous studies highlighted the importance of TLR/MyD88 signaling in B cells and DCs for the induction of antibodies *in vivo* (22, 25). Therefore, it was not as surprising to observe a decrease in the humoral response in the DC-MyD88^−/−^ and B-cell-MyD88^−/−^ mice in our experiments. This was especially evident in studies where MyD88 signaling in B cells *in vivo* was shown to be critical for induction of antibody-secreting cells upon vaccination (19), important for induction anti-retroviral GC responses (20) and required for induction of functional CD4 T cells producing IFN-γ to control IgG2c subclass production (21–23). Recent studies have shown that TLR-MyD88 signaling in DCs is required for induction of antibodies when immunized with vaccine containing soluble TLR9 ligands using DC-MyD88^−/−^ mice (27). Our observations of a decrease in T-cell-dependent antibody responses in DC-MyD88^−/−^ and B-cell MyD88^−/−^ mice with the use of different TLR ligand-based adjuvants were consistent with these previous published findings. However, the requirement of MyD88 signaling in macrophages for *in vivo* TLR ligand-based vaccine adjuvant activity has never been demonstrated or previously investigated.

An essential immunological process that is required for the production of significant amounts of high affinity T-cell-dependent antibodies is the generation of GCs. These are transient structures that form within secondary lymphoid organs in response to antigens (24) and are the main source of memory B cells, as well as plasma cells producing high levels of antigen-specific antibodies of different subclasses (53). In the current study, we have demonstrated that Mac-MyD88^−/−^ mice had a dramatic decrease in both GC formation and the percentage of GC B cells found after immunization with vaccines containing TLR ligand PorB or CpG as adjuvants. The diminished number and reduced area of GC cells is the probable reason why Mac-MyD88^−/−^ mice had significantly lower antigen-specific antibody levels upon vaccination with MyD88-dependent TLR ligand-based adjuvants, as compared to WT mice. As a control, we have shown that Mac-MyD88^−/−^ mice form normal GCs and antigen-specific antibody levels when the TLR-independent adjuvant alum was used in the vaccine formulation (or when sheep red blood cells are given), indicating that these mice are not inherently impaired in GC formation.

It is known that upon subcutaneous immunization, the content of the vaccine is partly taken up by the resident DCs or macrophages at the immunization site, which then traffic to the draining lymph nodes and spleen. The vaccine components can also journey to the draining lymph nodes via vitreous pressure. Within the secondary lymphoid organs, they encounter unique sets of macrophages (54) including metallophillic macrophages (MM, CD169^+^), MZM (MARCO^+^), red pulp macrophages (CD206^+^), and white pulp macrophages (CD68^+^, tingible body macrophages), with a variety of functions depending on their locations *in vivo* (54). MM CD169^+^ macrophages line the subcapsular sinus of the draining lymph nodes and surround the follicular B cells in the spleen; they have been shown to be able to capture antigens and present these antigens complexes directly to B cells and follicular DC (55). Interestingly, various investigators have shown that depletion of macrophages in the spleen resulted in an inability to form GCs (56, 57), but the role of MyD88 signaling in the adjuvant-related function of these cells has not been examined. In the set of presented studies, we have revealed a unique and, here-to-for, unexamined role of macrophage TLR/MyD88 signaling in TLR ligand-based adjuvant function, which cannot be rescued by normal DC or B cell function. This would suggest that the MyD88 defect in macrophages might be uniquely affecting MM and MZM function and the reason why these mice cannot form GCs when TLR ligands are used as vaccine adjuvants.

Previous studies have shown that mice with B cell-specific MyD88 deletion have a significant reduction in GC formation upon stimulation with endogenous nucleic acid recognized by TLR 9 or 7 in a lupus-like autoimmune disease mouse model in Lyn^−/−^ mice (58). Interestingly, even though we observed a decrease in OVA-specific antibody responses in the sera of OVA/PorB immunized B-cell-MyD88^−/−^ mice, we found no significant decrease in GC area. This suggests that intact MyD88 signaling in B cells is not required for GC formation when PorB is used as an adjuvant; however, it likely still required for a switch to plasma cells to allow for production of OVA-specific IgG. More studies are needed to investigate the decrease in antibody responses in B-cell-MyD88^−/−^ mice by examining antibody-secreting cells. Another study showed that TLR9 signaling in DCs (soluble TLR9) and B cells (virus-like particle associated TLR9) controls the magnitude of the GC reaction upon immunization with vaccine formulated with the TLR9 ligand, CpG (48). We observed a similar decrease in GC formation in DC-MyD88^−/−^ mice upon immunization with TLR/MyD88-dependent adjuvants but not with TLR-independent adjuvants.

There is evidence that alum adjuvant activity may be partially MyD88 dependent (47), and we do see a reduced antibody response in the three APC-MyD88^−/−^ mice strains as compared to WT mice when immunized with alum-formulated vaccines. However, the decrease in the antibody response was minimal when compared to the abrogation of antigen-specific antibodies with TLR ligand-based adjuvants in the MyD88 conditional KO mice. MF59 adjuvant activity has been shown to be MyD88 dependent (12); however, in our studies the antibody levels induced when MF59 was used as an adjuvant in the MyD88 conditional KO mice were not greatly affected. Therefore, it is most likely that MF59 adjuvant activity requires MyD88 signaling in non-hematologic cells (i.e., stromal cells), or more than one APC at a time. More studies are needed to identify the cell type or set of cell types that are responsible for its adjuvant activity.

In general, multiple cytokines have been shown to be involved in adjuvant mediated vaccine-induced immune responses (59–61). Here, we found that PorB, as a TLR2 ligand vaccine adjuvant, induces high serum levels of many cytokines known to be involved with efficacious vaccine responses, including pro-inflammatory cytokines (IL-1β and IL-6), chemokine (IP-10), Th2 type cytokines (IL-4, IL-5, and IL-13), and Th1 type cytokines (IL-12 and IFN-γ) in WT mice shortly after immunization. The selective deletion of MyD88 in macrophages resulted in a profound decrease in most of these effector molecules except for IL-6, IL-4, and IFN-γ, which remained unchanged. Adjuvants vary greatly in the character and intensity of induced responses (42), and this is reflected in the heterogeneity of the observed cytokine levels with the different types of adjuvant used in our study. Overall, our findings demonstrate that the examined adjuvants induced heterogeneous responses and that MyD88 signaling in each of these APCs affects the overall profile of cytokine induction induced by these adjuvanted OVA formulations. However, at this point, it is difficult to ascertain whether these alterations in cytokine profiles are directly related to the alterations in adjuvant activity and further work is necessary to clarify this process, especially in regard to B and T effector and memory cell induction and GC formation.

In summary, our major findings indicate that MyD88 signaling in APCs, especially in macrophages, is essential for PorB (TLR2 ligand) vaccine adjuvant activity. It also appears that the same can be said for CpG, a TLR9 ligand vaccine adjuvant, but this is not true for TLR ligand-independent adjuvants like alum or MF59. These new insights could aid in vaccine development by allowing more intelligent and judicious use of these and other vaccine adjuvants to target specific cell types and induce specific type of responses. Vaccines containing TLR ligand-based adjuvants can be formulated and administered in a specific way so that they target specific APC types in the lymphoid organs and engage specific TLR or sets of TLRs in order to enhance types of immune responses needed for protection.

## Ethics Statement

This study was carried out in accordance with the Guide for the Care and Use of Laboratory Animals of the National Institutes of Health approved by the Institutional Animal Care and Use Committee (IACUC) of the Boston University School of Medicine. The protocol was approved by the Boston University School of Medicine IACUC.

## Author Contributions

MM and LW designed research; MM performed research; MM, MR, and LW analyzed data and wrote the paper.

## Conflict of Interest Statement

The authors declare that the research was conducted in the absence of any commercial or financial relationships that could be construed as a potential conflict of interest.
