# Supplementary material for: Toll-Like Receptor Ligand-Based Vaccine Adjuvants Require Intact MyD88 Signaling in Antigen-Presenting Cells for Germinal Center Formation and Antibody Production
Source: Front Immunol. 2017 Mar 3;8:225. doi: 10.3389/fimmu.2017.00225 (PMC5334362; doi:10.3389/fimmu.2017.00225)
Supplement: Supplementary file 1 [file Presentation_1.pdf]

frontiers

Supplementary Material

Article Title

M. Mosaheb, M. Reiser, L.M. Wetzler*

*** Correspondence:** Corresponding Author: lwetzler@bu.edu

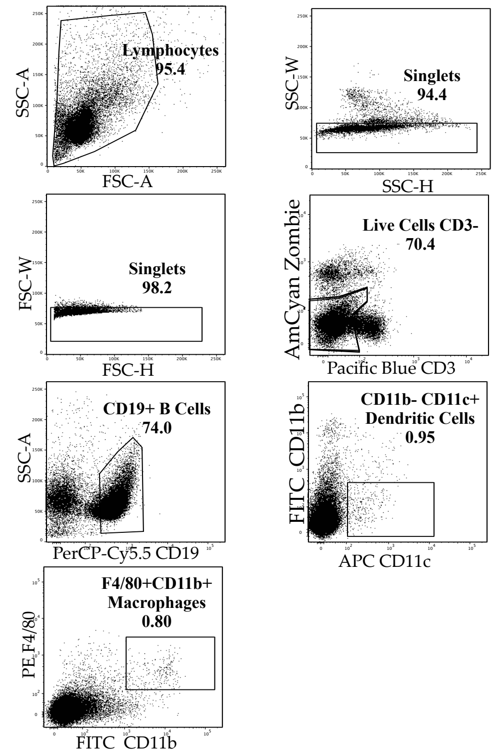

Supplementary Figure S1. Gating strategies for flow cytometry analysis for sorting of B-cells (CD3-CD19+), DCs (CD11b-CD11c+) and macrophages (CD11b+F4/80+). Spleens from WT, B-Cell-MyD88-/-, DC-MyD88-/- and Mac-MyD88-/- mice were harvested, single cells suspensions were prepared and red blood cells lysed. The splenocytes were analyzed with live/dead stain (Zombie Aqua viability kit), Pacific Blue anti-CD3, PerCP-Cy5.5 anti-CD19, FITC anti-CD11b, APC anti-CD11c, and PE anti-F4/80 fluorescently labeled antibodies all purchased from Biolegend. B-cells (CD3-CD19+), DCs (CD11b-CD11c+) and macrophages (CD11b+F4/80+) were sorted on a FACS ARIA II SORP Cell Sorter by their specific cell markers. Dead cells and CD3+ cells were gated out. There were two mice per genotype examined.

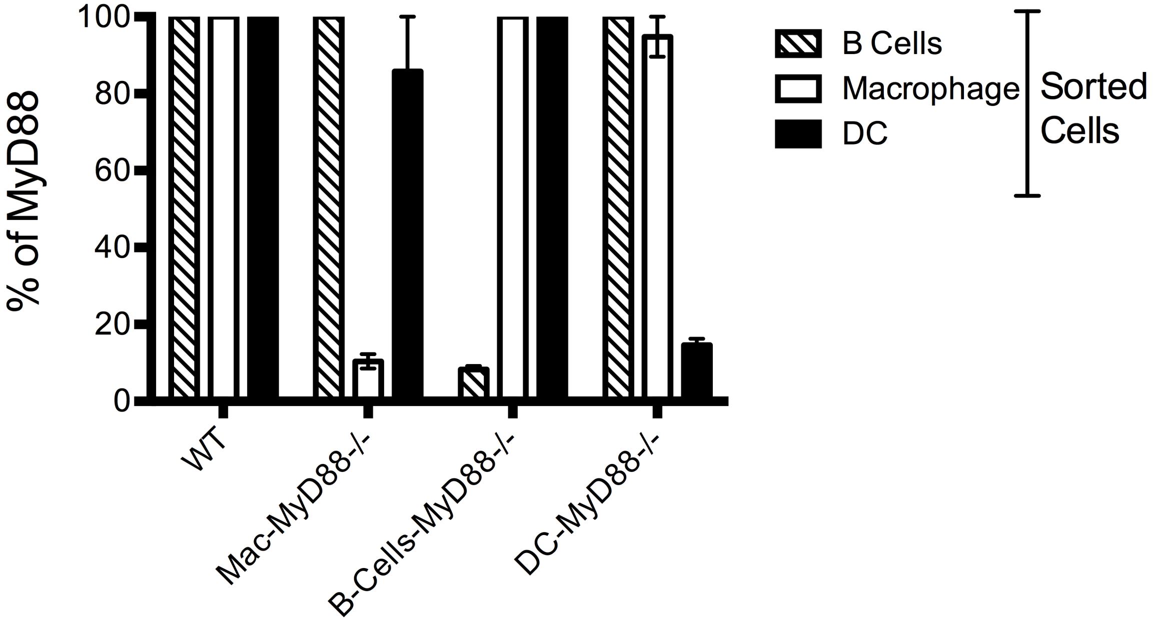

Supplementary Figure S2. MyD88 DNA expression in specific cell types. Amount of MyD88 deletion in various cell types purified from spleens was determined by measuring the amount of residual MyD88. DNA from samples sorted as described in Figure S1 was isolated using DNeasy DNA isolation kit (Qiagen, Hilden, Germany) and quantitated on Nanodrop 2000 (Thermo Scientific, USA). Using quantitative real time PCR (qRT-PCR) (Qiagen SYBR Green kit) with the ABI Prism 7000 Sequence Detection System, the amount of residual MyD88 was measured in B-cells (CD3-CD19+), macrophages (CD3-CD11b+F4/80+) and dendritic cells (CD3-CD11b-CD11c+). There were two mice per group and this was performed twice. The qRT-PCR analysis was done with the threshold cycle (Ct) of MyD88 gene normalized to β-actin (internal control) by the Livak method. Each test sample was also normalized to the WT samples (calibrator). MyD88 deletion efficacy was calculated where 2- ΔΔCt gives us the fold change normalized to the internal control and calibrator sample. Percent deletion was calculated.

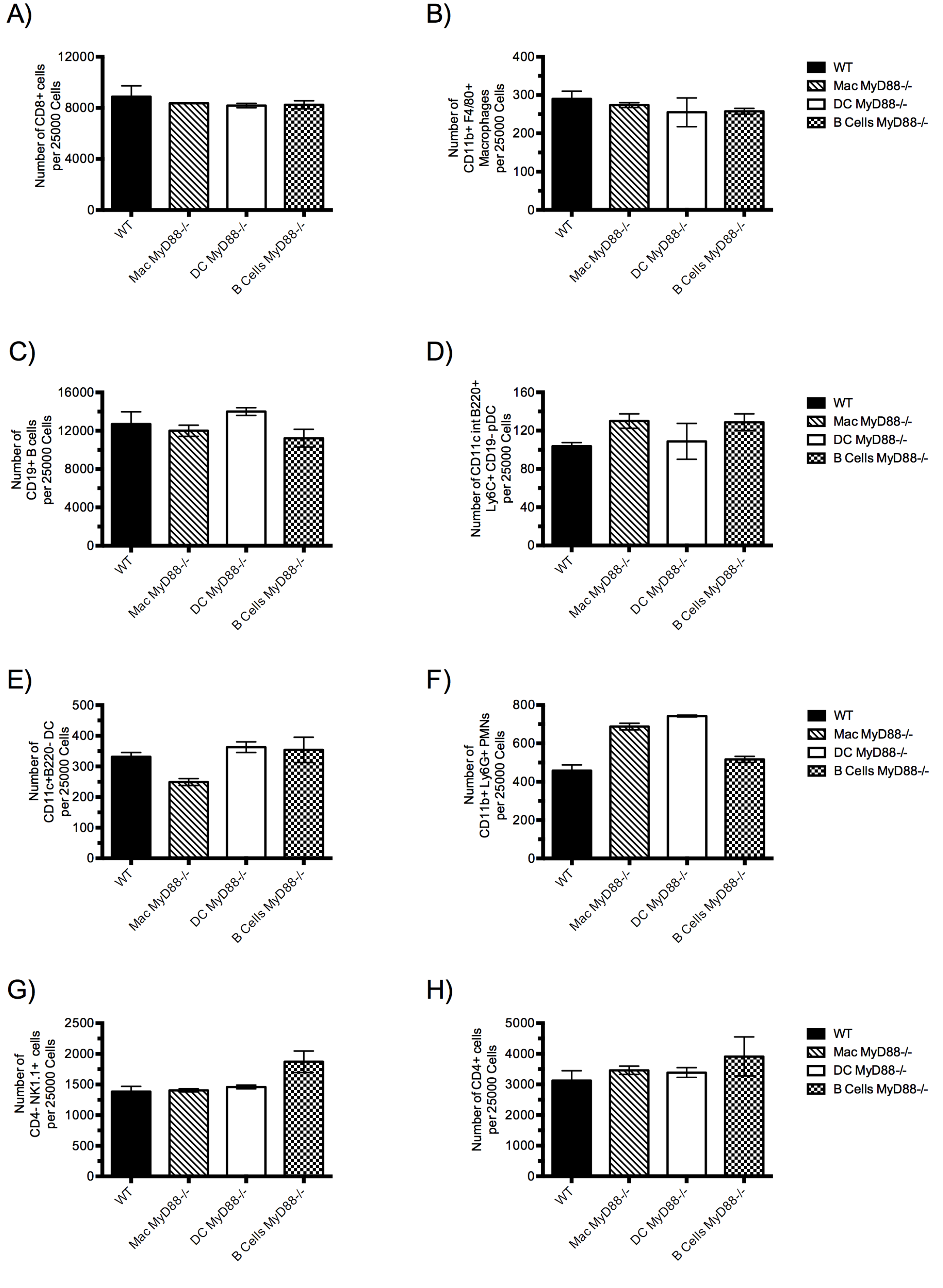

Supplementary Figure S3. Deletion of MyD88 in DCs, macrophages or B-cells did not affect the different cell populations in the mouse spleen. Spleens from WT, B-Cell-MyD88-/-, DC-MyD88-/- and Mac-MyD88-/- mice were harvested, single cells suspensions were prepared and red blood cells lysed. Splenocytes were stained with fluorescently labeled antibodies and the number of **A)** CD8+ T-cells, **B)** CD11b+F4/80+ Macrophages, **C)** CD19+ B cells, **D)**, CD11c int B220+ Ly6C+ CD19- pDCs **E)** CD11c+B220- DCs, **F)** CD11b+Ly6G+ PMNs, **G)** NK1.1+CD4- T-cells and H) CD4+ T-cells were measured by Flow Cytomteric analysis. There were two mice per genotype.

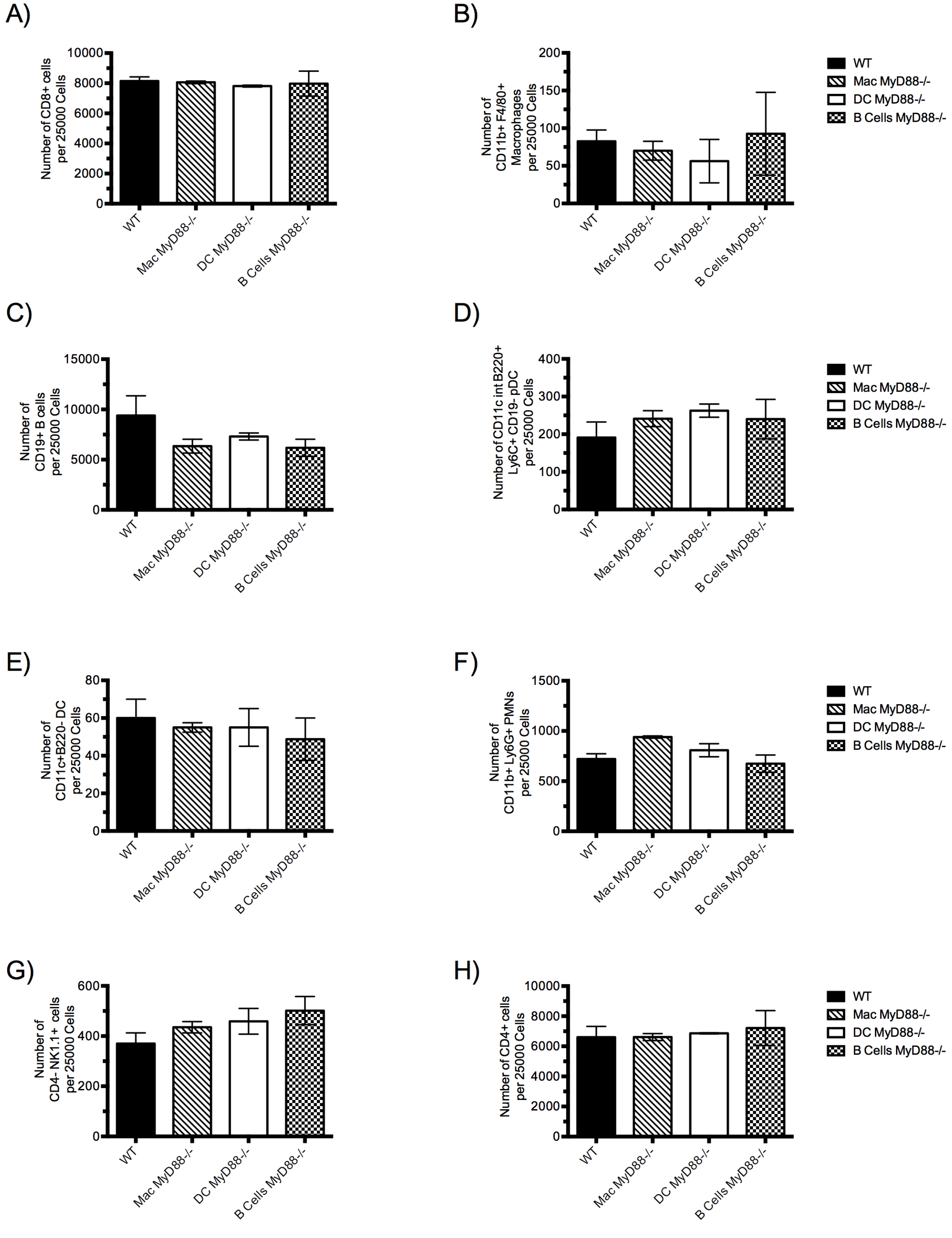

Supplementary Figure S4. Deletion of MyD88 in DCs, macrophages or B-cells did not affect the different cell populations in the mouse inguinal, axillary and brachial lymph nodes combined.
Single cell suspensions from these 6 draining lymph nodes were prepared and stained with fluorescently labeled antibodies and the number of **A)** CD8+ T-cells, **B)** CD11b+F4/80+ Macrophages, **C)** CD19+ B cells, **D)** CD11c int B220+Ly6C+CD19- pDCs, **E)** CD11c+B220- DCs, **F)** CD11b+Ly6G+ PMNs, **G)** NK1.1+CD4- T-cells and H) CD4+ T-cells were measured by Flow Cytomteric analysis. There were two mice per mouse genotype analyzed.
